# Development of Sodium Alginate/Cellulose Nanofiber (SA/CNF)-Based Hydrogels for Enhancing Probiotic Stability

**DOI:** 10.3390/gels12060491

**Published:** 2026-06-02

**Authors:** Hyeon Ji Jeon, Bo Yeong Park, Ju Hyun Min, Gyu Ri Shin, Hye Min Jeong, Kwang Yong Seol, Ju-Hoon Lee, Younghoon Kim, Jungwoo Yang, Young Hoon Jung

**Affiliations:** 1 School of Food Science and Biotechnology, Food and Bio-Industry Institute, Kyungpook National University, Daegu 41566, Republic of Korea; hhyyeeoonnji@knu.ac.kr (H.J.J.); pho4028@naver.com (B.Y.P.); 2021knu@naver.com (J.H.M.); gyul00824@gmail.com (G.R.S.); jeonghyemin0534125@gmail.com (H.M.J.); esabil58@gmail.com (K.Y.S.); 2 Department of Agricultural Biotechnology, Research Institute of Agriculture and Life Science, Seoul National University, Seoul 08826, Republic of Korea; juhlee@snu.ac.kr (J.-H.L.); ykeys2584@snu.ac.kr (Y.K.); 3 Department of Microbiology, College of Medicine, Dongguk University, Gyeongju 38066, Republic of Korea; 4 Department of Food Science and Biotechnology, Universitas Brawijaya, Malang 65145, Indonesia

**Keywords:** hydrogels, probiotics, delivery system, cellulose nanofibers, sodium alginate

## Abstract

Probiotics can promote gut health, but their efficacy is often limited by low viability and metabolic activity in the gastrointestinal (GI) tract. This study aimed to develop protective hydrogels for encapsulating *Lactiplantibacillus plantarum* CJLP 133 using a composite matrix of sodium alginate (SA) and cellulose nanofibers (CNFs). *L. plantarum* CJLP 133-loaded hydrogel beads were fabricated via the ionic gelation technique using an optimized formulation of SA and CNF. Scanning electron microscopy revealed that CNF integration improved spherical morphology with reduced surface cracking. Fourier transform infrared spectroscopy confirmed the formation of intermolecular hydrogen bonds between SA and CNF. CNF integration also reduced gumminess and chewiness, resulting in a softer texture. The survival rate of *L. plantarum* CJLP 133 remained high following thermal exposure and freeze-drying. The in vitro GI delivery system demonstrated a protective swelling profile in stimulated gastric fluid and a targeted, highly efficient release profile in stimulated intestinal fluid. Finally, the 3% SA + 0.5% CNF hydrogel with *L. plantarum* CJLP 133 exhibited significant synbiotic effects, enhancing probiotic growth, intestinal adhesion, and butyrate and succinate production. These results suggest that the SA/CNF-based hydrogel is an effective delivery system that ensures the targeted release of probiotics within the GI tract.

## 1. Introduction

Probiotics are living microorganisms that provide health benefits to the host when consumed in adequate amounts [1]. Probiotic products must maintain a minimum viable cell count and retain their activity throughout storage, transportation, and digestion [2]. Many studies have examined the encapsulation of probiotics to improve their viability and stability [3,4,5], particularly through their incorporation into hydrogel matrices [6,7]. Hydrogels are three-dimensional (3D) structures consisting of cross-linked hydrophilic polymers [8]. Given their inherent physicochemical properties, they have been extensively used as promising matrices for probiotic encapsulation [9,10]. In particular, their high water retention capacity effectively mimics the physiological conditions necessary for microbial growth, thereby improving survival [11]. Moreover, the biocompatible polymeric matrix acts as a protective buffer that shields probiotics from osmotic stress and mechanical shear during storage [12,13].

Sodium alginate (SA)-based hydrogels are among the most widely used biopolymers for probiotic encapsulation because of their biocompatibility, nontoxicity, and mild gelation process [14]. SA can easily form hydrogels through ionic crosslinking with divalent cations, particularly calcium ions (Ca^2+^), resulting in a characteristic “egg-box” structure within the polymer network [15,16]. This egg-box configuration creates a stable, dense gel matrix that effectively entraps bacterial cells, further enhancing their viability during manufacturing and storage [17]. Furthermore, given the pH-responsive behavior of SA, these hydrogels can protect the cargo from harsh gastrointestinal (GI) conditions while facilitating site-specific release within the intestinal environment [18,19,20]. However, despite these functional advantages, SA-based hydrogels possess inherent structural vulnerabilities that limit their efficacy as a standalone encapsulation matrix [21,22,23]. The intrinsic porosity of these hydrogels makes them susceptible to excessive swelling or structural erosion, resulting in the unintended leakage of encapsulated probiotics [24]. Such premature release of probiotics can diminish probiotic viability when exposed to external stressors. To overcome these limitations, the incorporation of nanocellulose into SA-based hydrogels has been proposed as an effective strategy for enhancing structural stability and protective performance.

Cellulose nanofibers (CNFs) are a type of nanocellulose from lignocellulosic biomass with a carbon content of more than 50%. They are extracted from plant-based biomass through fibrillation processes and consist of highly ordered, lengthy crystalline and amorphous regions [25]. Given their high degree of crystallinity, surface area, and strength [26], cellulose-based hydrogels have been widely employed as physical patches for wound healing and drug delivery systems and have also been explored as ionogels or metagels for use in device-oriented technologies [27,28,29,30,31]. CNF-based hydrogels primarily formed physical interactions rather than ionic crosslinking; they often exhibit limited responsiveness to environmental conditions [32,33,34]. Additionally, CNF-based hydrogels exhibit a more open fibrillar structure [35]; thus, the combination of SA and CNF can enhance mechanical properties, allowing the composite to serve as an excellent filtration or film matrix [36,37]. Prior studies have reported high stability of SA-based hydrogels incorporated with CNF, resulting from interactions with the hydroxyl and carboxyl groups present on the hydrogel surface [38,39]. In addition, several studies have explored the use of SA/cellulose composite hydrogels for probiotic encapsulation, demonstrating their potential for improving bacterial survival within the gastrointestinal (GI) tract [40,41]. However, the potential as a synbiotic delivery system remains largely underexplored, particularly in terms of integrating both protective and functional roles under GI conditions.

In this study, an SA/CNF composite formed hydrogel beads to improve the viability and activity of the probiotic *Lactiplantibacillus plantarum* CJLP 133 under environmental conditions. In particular, the structural and physicochemical characteristics of the SA/CNF hydrogel beads, including their morphology, chemical interactions, and textural properties, were comprehensively analyzed to confirm successful bead formation. After determining the encapsulation efficiency, the functional properties and controlled release potential of the encapsulated probiotic strain were evaluated under simulated GI conditions. Finally, the prebiotic effects were assessed to determine the feasibility of these SA/CNF hydrogels as a synbiotic encapsulation system for enhanced probiotic delivery. Consequently, this study demonstrates that the SA/CNF encapsulation strategy can significantly improve the GI survival and colonization of probiotics, thereby contributing to more effective probiotic delivery systems.

## 2. Results and Discussion

### 2.1. Physicochemical and Structural Characteristics of SA/CNF Hydrogel Beads

#### 2.1.1. Morphological Analysis of SA/CNF Hydrogel Beads

As the size, shape, and surface morphology of hydrogel beads are crucial for ensuring stable probiotic delivery and consumer acceptance [17], the physicochemical properties of the SA/CNF hydrogel beads were evaluated in this study.

The diameters of hydrogel beads under different formulation conditions are presented in Table 1. In particular, the synergistic effects of varying concentrations of SA (1%, 2%, and 3%), CNFs (0.5% and 1%), and the cross-linking agent calcium chloride anhydride (CaCl_2_; 0.1 M and 0.5 M) on the formation of stable hydrogel beads were investigated. The use of a dash (-) means the SA/CNF mixture failed to form the hydrogel beads. The diameters of all prepared hydrogel beads were consistently below 3 mm. This size is considered acceptable for oral consumption and does not cause ingestion discomfort [10]. The diameters of the hydrogel beads ranged from 1.24 ± 0.08 to 2.55 ± 0.02 mm, depending on the concentration and ratio of SA and CNF. SA concentration had a substantial effect, with higher concentrations resulting in significantly larger beads. Furthermore, the incorporation of CNF resulted in a synergistic increase in size; however, this effect was modulated by the SA content. Although the CNF concentration did not exhibit a distinct trend at 1% SA, it significantly increased bead size in formulations containing 2% and 3% SA. Higher SA concentrations enhanced the viscosity of the feed solution, facilitating intensive molecular entanglement within the alginate network and subsequently resulting in the formation of SA-based hydrogel beads with a larger particle size [42]. Although hydrogel beads formed in 0.1 M CaCl_2_ were slightly larger than those formed in 0.5 M CaCl_2_, no significant difference was noted between the two concentrations. SA can form a gel upon contact with a CaCl_2_ solution. This occurs due to the interaction of divalent calcium ions with the carboxylate groups of SA, leading to ionic crosslinking between polymer strands. This “egg-box” model forms a three-dimensional network structure of gels [15,43]. Therefore, a lower concentration of CaCl_2_ (0.1 M) was selected for subsequent experiments to improve economic efficiency.

**Figure 1:**
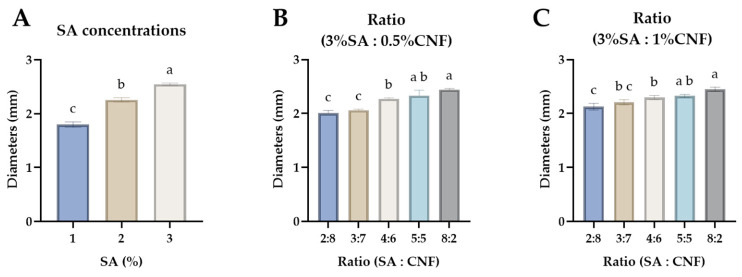
Diameter (mm) of (**A**) Sodium alginate (SA)-based hydrogel beads in formulations containing 1%, 2%, and 3% SA. (**B**) SA/Cellulose nanofiber (CNF) hydrogel beads with a 3% SA to 0.5% CNF ratio. (**C**) SA/CNF hydrogel beads with a 3% SA to 1% CNF ratio. Data are presented as the mean ± SD (*n* = 6). Different lowercase letters indicate significant differences (*p* < 0.05).

After the diameters of the hydrogel beads were visually compared based on the concentrations or ratios of SA and CNF, the formulation with the largest diameter was selected for subsequent analyses to maximize the loading number of probiotics per capsule while maintaining the diameter within 3 mm—a size safe for ingestion (Figure 1). The 3% SA-based hydrogel beads sized 2.55 ± 0.02 mm were selected because they produced the largest bead size. Therefore, while other parameters, such as CNF concentrations and the ratio of SA to CNF, varied, the SA concentration was held constant at 3%. The diameter of the hydrogel beads was dependent on the mixing ratio, with an increase in diameter noted as the proportion of SA relative to CNF increased. As all prepared beads met the size requirement for oral ingestion, three formulations exhibiting the maximal diameters (3% SA, 8:2 mixture of 3% SA with 0.5% CNF, and 8:2 mixture of 3% SA with 1% CNF) were chosen for further analyses to ensure an efficient payload of the encapsulated strain.

**Figure 2:**
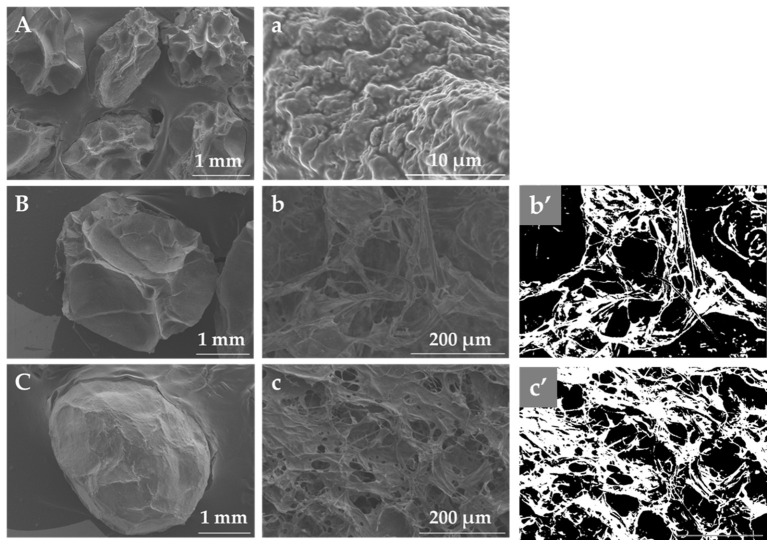
Surface images of SA/CNF hydrogel beads. (**A**,**a**) 3% SA, (**B**,**b**,**b′**) 3% SA + 0.5% CNF, and (**C**,**c**,**c′**) 3% SA + 1% CNF. (**A**–**C**) present the structure of the whole beads (30×), while (**a**–**c**) present the enlarged surface structure of the beads (3000×). Images (**b′**,**c′**) were used for quantitative pore size analysis using ImageJ software.

The surface morphology of SA/CNF hydrogel beads was observed using scanning electron microscopy (SEM), with low-magnification images (30×) showing the overall spherical shape and high-magnification images (3000×) showing microstructural features (Figure 2). While the 3% SA hydrogel produced wrinkled and less porous beads, the incorporation of CNF (0.5% and 1%) resulted in a more spherical and plump morphology. Moreover, 1% CNF resulted in more well-defined spherical shapes than 0.5% CNF, likely due to the reinforced structural integrity of higher concentrations of the CNF network [44]. In the low-magnification images, pores resulting from the incorporation of CNF were observed; hydrogels with higher CNF concentrations exhibited a finer porous structure. Specifically, the average pore diameters were measured from SEM images using ImageJ software by analyzing over 400 pores per sample, excluding the maximum and minimum values. The pore size decreased from 28.98 ± 43.86 um for 3% SA + 0.5% CNF hydrogels to 11.04 ± 14.65 um for 3% SA + 1% CNF hydrogels, indicating the formation of smaller pores at the higher CNF concentrations. The formation of a refined porous structure has been frequently reported in CNF-based hydrogels [45,46], and the decrease in pore size at higher CNF concentrations may be attributed to the formation of a more compact internal network structure during hydrogel formation.

#### 2.1.2. Textural Properties of SA/CNF Hydrogel Beads

Texture profile analysis (TPA) was performed to assess the mechanical integrity of the SA/CNF hydrogel beads (Table 2), as the structural properties of a hydrogel are crucial for protecting the encapsulated probiotics from processing stress and GI transit and for meeting consumer preferences [47,48]. The 3% SA + 0.5% CNF hydrogel showed the lowest value across all TPA parameters, including hardness, springiness, gumminess, chewiness, and cohesiveness. In particular, the hardness, gumminess, and chewiness of 3% SA + 0.5% CNF hydrogels were significantly lower (1.23-, 1.27-, and 1.31- fold, respectively) than those of 3% SA hydrogels with *p* < 0.001. These results indicate that 3% SA + 0.5% CNF hydrogels have high potential for food applications, as they exhibit minimal tolerance to mechanical deformation because of favorable textural properties, such as hardness, gumminess, and chewiness. Low levels of gumminess and chewiness can offer a smooth texture and improve mouth feel during oral consumption [49]. However, no significant differences were noted in the levels of adhesiveness, springiness, or cohesiveness when CNF was added. This suggests that CNF did not significantly alter the inherent textural properties of the SA matrix, indicating the potential for the efficient release of encapsulated probiotics due to the high flexibility of SA.

#### 2.1.3. Chemical Interactions in SA/CNF Hydrogel Beads

**Figure 3:**
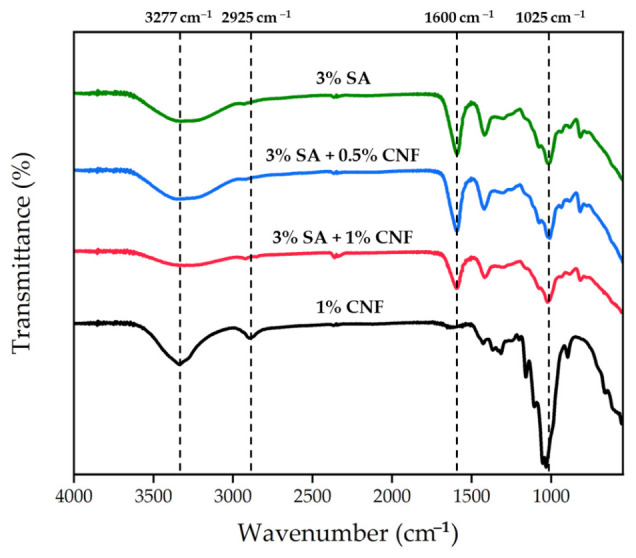
Fourier transform infrared (FT-IR) spectra of pure 3% SA and 1% CNF hydrogels and SA/CNF composite hydrogels with varying CNF concentrations (0.5% and 1%).

The chemical interactions in SA/CNF hydrogel beads were analyzed by Fourier transform infrared (FT-IR) spectroscopy (Figure 3). The incorporation of SA and CNF induced minor variations in the specific peaks of the hydrogels to varying degrees. In particular, the characteristic carboxyl group (-COO) peaks of SA were observed at 1410–1600 cm^−1^ in all SA-based hydrogel samples [50]. The 1% CNF sample exhibited a distinct peak at 2925 cm^−1^, corresponding to the C–H stretching vibrations of the cellulose backbone [51]. This peak was also observed in the SA/CNF hydrogel beads, although with reduced intensity. Moreover, the characteristic peak at 1025 cm^−1^ observed in the 1% CNF sample was detected in the SA/CNF hydrogels, indicating the presence of CNF in the SA network [52]. The absorption band at around 3277 cm^−1^ is attributed to O-H stretching vibrations in the SA/CNF composite gels, and no significant peak shift was observed among the samples, suggesting limited changes in the hydrogen-bonding [53].

Notably, no new peaks resulting from the formation of new chemical interactions were detected during the formation of the SA/CNF complex. This phenomenon indicated that the bonding between SA and CNF did not involve the formation of new covalent bonds. Instead, it can be explained by physical interactions such as hydrogen bonding and physical entanglement [54]. Therefore, SA/CNF hydrogels can exhibit combined properties of both components without substantial chemical modification.

### 2.2. Stability of L. plantarum CJLP 133 Encapsulated in SA/CNF Hydrogel Beads

Based on the textural profiles and chemical interactions of the SA/CNF composite, *L. plantarum* CJLP 133-loaded beads were fabricated to assess the feasibility of SA/CNF hydrogels as a probiotic delivery system. *L. plantarum* CJLP 133 exhibits probiotic properties and several health benefits, such as the alleviation of atopic dermatitis and allergic rhinitis [55,56,57,58]. Therefore, stable encapsulation within SA/CNF hydrogel beads is essential for targeted delivery and utilization of its therapeutic effects.

The encapsulation efficiency of *L. plantarum* CJLP 133 was evaluated to confirm its potential for probiotic stabilization (Table 3). All SA/CNF hydrogels had a high encapsulation efficiency of over 95%. Compared with previous reports of alginate-based encapsulation efficiencies ranging from 80% to 90% [59,60], the SA/CNF hydrogel beads exhibited a stable probiotic loading capacity. The consistently high efficiency with both 0.5% and 1% CNF concentrations suggests that CNF did not compromise the stable encapsulation efficiency of the SA-based hydrogels.

**Figure 4:**
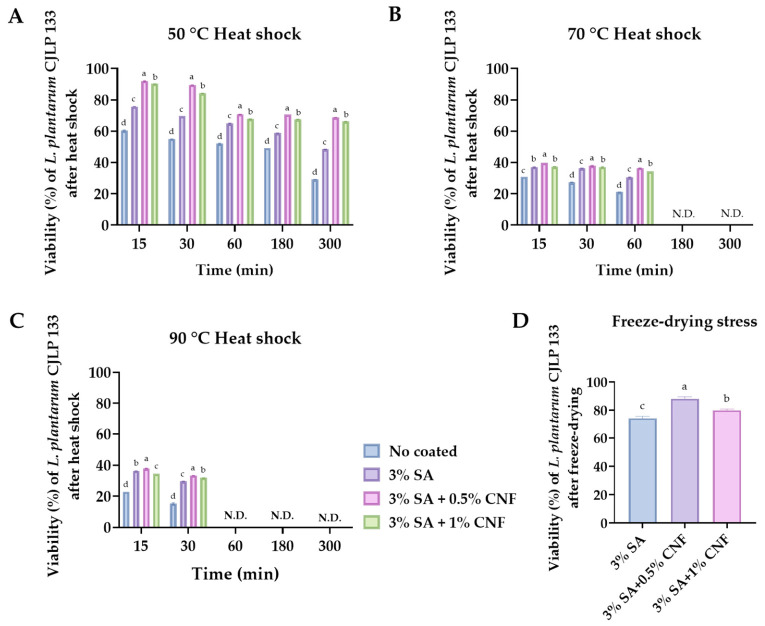
Survival rate and stability of *L. plantarum* CJLP 133 under thermal stress and freeze-drying. Survival rate (%) of *L. plantarum* CJLP 133 after heat treatment at (**A**) 50 °C, (**B**) 70 °C, and (**C**) 90 °C. (**D**) Viability after freeze-drying. Data are presented as the mean ± SD (*n* = 3). Different lowercase letters indicate significant differences (*p* < 0.05).

Next, the viability of *L. plantarum* CJLP 133 encapsulated in SA/CNF hydrogel beads was assessed after heat shock and freeze-drying to determine whether the hydrogel protects the entrapped probiotics during manufacturing (Figure 4). The 3% SA + 0.5% CNF hydrogel exhibited the highest probiotic survival under thermal stress at 50 °C, 60 °C, and 90 °C (Figure 4A–C). Compared with the initial 15 min exposure, the survival rate of uncoated cells decreased by 51.83% after 300 min at 50 °C. In contrast, the viability of the cells encapsulated in 3% SA + 0.5% CNF hydrogels decreased by only 25.30%. Furthermore, heat shock at 60 °C reduced the viability of uncoated cells by 31.80% within 15–60 min of exposure. However, the 3% SA + 0.5% CNF hydrogel protected the cells, limiting the reduction to only 8.66%. While heat shock at 90 °C killed all probiotic cells after over 60 min, the viability of uncoated cells decreased by 32.53% during 15–30 min of exposure at 90 °C; however, the 3% SA + 0.5% CNF hydrogel-coated cells exhibited only a 12.06% reduction.

Freeze-drying is a widely used process for manufacturing probiotic products [61]. The highest viability (88.18 ± 1.32%) was noted in 3% SA + 0.5% CNF hydrogel beads (Figure 4D). The survival rate of *L. plantarum* CJLP 133 was 74.05 ± 1.56% and 79.96 ± 0.88% in 3% SA and 3% SA + 1% CNF hydrogels, respectively. This is because hydrogen bonding within CNF can protect the entrapped cells by inducing pore shrinkage after freeze-drying due to the high surface tension of water [62]. Probiotic viability beyond 85% suggests that SA/CNF hydrogels are suitable for freeze-dried formulations, making their storage and transportation convenient [63].

These results suggest that SA/CNF hydrogels can effectively preserve entrapped probiotic viability under high-temperature and freeze-drying processes, with the 3% SA + 0.5% CNF formulation providing the most stable protection. Nevertheless, further studies are needed to determine long-term storage stability despite the demonstration of protectability against acute heat shock and freeze-drying. These aspects will be addressed in subsequent investigations to enhance the industrial application.

### 2.3. Characteristics of SA/CNF Hydrogels for Targeted Delivery

#### 2.3.1. Acid and Bile Tolerance of SA/CNF Hydrogels

**Figure 5:**
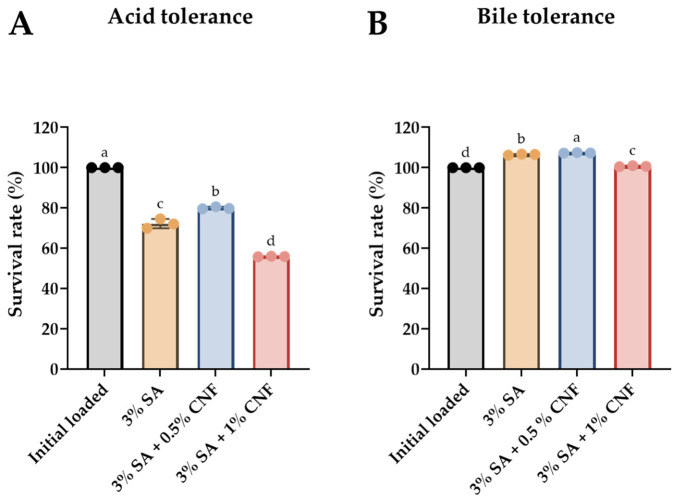
The survival rate (%) of *L. plantarum* CJLP 133 in SA/CNF hydrogel beads under (**A**) 2.5 pH and (**B**) 0.3% bile salt medium. Data are presented as the mean ± SD (*n* = 3). Different lowercase letters indicate significant differences (*p* < 0.05).

Probiotic encapsulation must maintain bacterial viability during exposure to low pH and bile salts, ensuring controlled release within the GI tract [64]. In this study, *L. plantarum* CJLP 133 encapsulated in 3% SA + 0.5% CNF hydrogels exhibited the highest viability, reaching 80.00 ± 0.46% and 107.37 ± 0.12% under acidic (pH 2.5) and bile salt conditions, respectively (Figure 5). However, the acid and bile tolerance capacities of the 3% SA + 1% CNF hydrogels were lower than those of the pure 3% SA hydrogels. In particular, the viability in the 3%SA + 1% CNF was only 55.92 ± 0.11% under acidic conditions and 100.75 ± 0.23% under bile salt conditions. On the other hand, 3% SA hydrogels maintained higher viability of 72.20 ± 1.88% and 106.51 ± 0.21% under acidic and bile salt conditions, respectively.

As CNF exhibits aggregative properties at pH levels below 3, excessive CNF under acidic conditions could induce self-aggregation, resulting in the formation of low-strength hydrogels [65,66]. Therefore, the decrease in protective efficacy at a higher CNF concentration (1%) may be related to a concentration-dependent threshold for strengthening the gels. Thus, 0.5% CNF seems optimal for protecting cells against low pH by producing physically stable hydrogels with SA. The bile tolerance of the encapsulated probiotics did not change significantly depending on the CNF concentration. These results indicate that the observed effects are related to acid tolerance rather than bile tolerance. These findings are consistent with those of previous studies on the use of CNF to protect probiotics [67].

#### 2.3.2. Controlled Release of SA/CNF Hydrogels in Simulated GI Conditions

**Figure 6:**
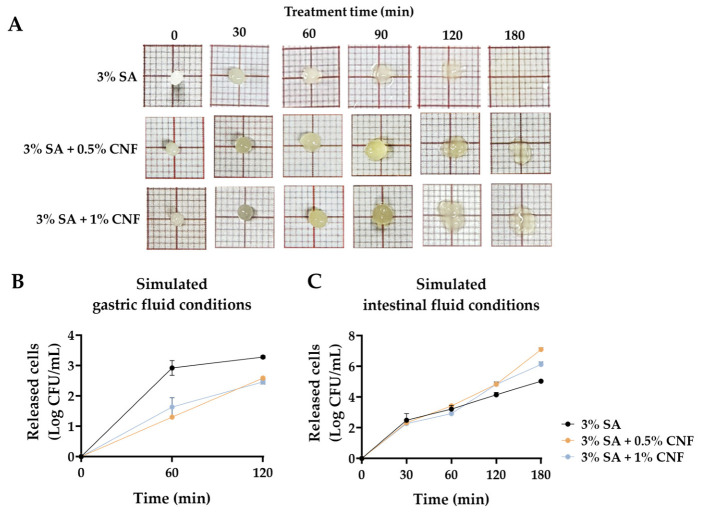
Swelling behavior and in vitro GI release profiles of SA/CNF hydrogel beads. (**A**) Photographic observation of the swelling and disintegration behaviors of SA/CNF hydrogel beads in SIF. Release profiles of encapsulated *L. plantarum* CJLP 133 under (**B**) SGF and (**C**) SIF conditions. Data are presented as the mean ± SD (*n* = 3).

Controlled release is one of the most important properties of probiotic encapsulation for targeted delivery in the host [68]. For controlled release, the encapsulation system should retain probiotic cells in encapsulation agents and not release them under simulated gastric fluid (SGF) conditions; however, it should release probiotic cells for colonization under stimulated intestinal fluid (SIF) conditions [69]. Hence, the swelling behavior of hydrogels and the released cell count were measured in SGF for 120 min and SIF for 180 min (Figure 6) [10]. The swelling behavior of the SA/CNF hydrogels in SIF was initially monitored visually to observe morphological transition over 180 min (Figure 6A). As shown in the figure, the 3% SA hydrogel beads exhibited swelling for up to 60 min and completely disintegrated after 180 min. The two CNF-containing hydrogel beads remained swollen for a longer period (up to 90 min) and then disintegrated after 180 min. However, they retained their spherical capsule shape better than the 3% SA hydrogel beads at the final time. It was difficult to distinguish the swelling behavior of the 0.5% CNF and 1% CNF samples by visual inspection.

Next, released cell counts were performed to accurately assess controlled release (Figure 6B,C). Compared with the pure SA hydrogel, the incorporation of CNF into the SA matrix improved controlled release by effectively inhibiting the premature leakage of probiotics in SGF while facilitating a more efficient release in SIF. In particular, after 120 min of exposure to SGF, the 3% SA + 0.5% CNF hydrogel and 3% SA + 1% CNF hydrogel exhibited 4.9- (*p* = 0.0003) and 6.61- fold (*p* = 0.0011) lower *L. plantarum* CJLP 133 release levels, respectively, than the 3% SA hydrogel. During the initial phase of SIF exposure (until 60 min), the number of released cells was similar across all formulations. However, a significant difference was noted at the final 180 min of SIF exposure. While the 3% SA + 1% CNF hydrogel exhibited a 13-fold (*p* < 0.0001) higher cell release than the 3% SA hydrogel, the 3% SA + 0.5% hydrogel exhibited a remarkably higher released cell count (114.82-fold) (*p* < 0.0001) than the pure 3% SA hydrogel. In addition, in SGF, the number of released cells from the 3% SA + 0.5% CNF and 3% SA + 1% CNF hydrogels showed no significant difference (*p* = 0.2467). However, in SIF, the 0.5% CNF formulation exhibited significantly higher cell release compared to the 1% CNF hydrogel (*p* = 0.0486).

The swelling behavior of a hydrogel is highly pH-dependent and related to the characteristics of each matrix [18]. The improved controlled release performance of the SA/CNF hydrogel beads can be primarily attributed to the unique structural properties of CNF, including its high surface area and fibrous morphology. These characteristics may provide a robust physical barrier against gastric enzymes and acidity. As shown in Figure 3, the incorporation of CNF into the SA hydrogel matrix promoted intermolecular hydrogen bonding. This interaction of CNF and SA reduced the space occupied by free water within the network, facilitating the formation of a dense and compact network [70]. In contrast, at high pH levels, the carboxyl residues of alginate were ionized, resulting in electrostatic repulsion between the polymer chains. This process, coupled with the exchange of Ca^2+^ for Na^+^, induced swelling and subsequent matrix disintegration, improving the controlled release of *L. plantarum* [71].

Although the SA/CNF formulation demonstrated controlled release and stability in the simulated GI tract, the current static model provides initial evidence of GI survival potential but does not replicate the dynamic nature of human digestion. Thus, the efficacy of the delivery system under dynamic conditions remains to be further established.

Interestingly, 0.5% CNF was consistently found to be the optimal concentration across all experimental parameters, providing the most effective balance of protection and release of *L. plantarum* CJLP 133. Based on these results, the 3% SA + 0.5% CNF formulation was compared with each matrix for subsequent evaluation of synbiotic efficacy.

### 2.4. Synbiotic Effects of L. plantarum CJLP 133 in SA/CNF Hydrogels

#### 2.4.1. Prebiotic Effects of *L. plantarum* CJLP 133 in SA/CNF Hydrogels

**Figure 7:**
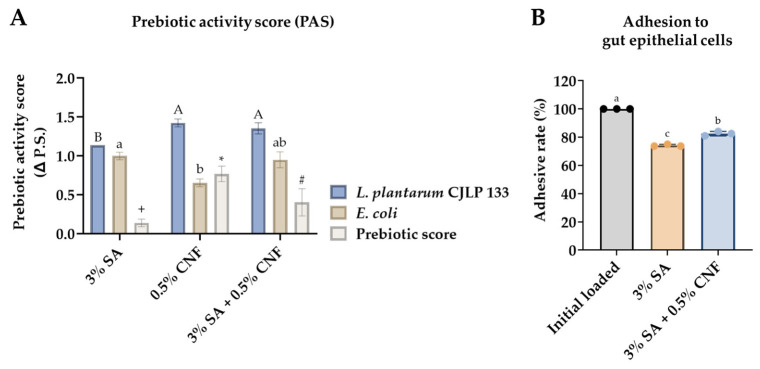
(**A**) Prebiotic activity score of SA/CNF hydrogels compared with SA or CNF alone. (**B**) Adhesion ability of SA/CNF hydrogels to gut epithelial cells. Data are presented as the mean ± SD (*n* = 3). Different letters indicate significant differences (*p* < 0.05) within the same category (uppercase letters, lowercase letters, and special characters [*, #, +], respectively).

To evaluate the potential of SA/CNF hydrogel beads as novel synbiotic products, their prebiotic activity score (PAS) and in vitro gut adhesion were determined (Figure 7). The PAS was calculated based on the differential growth of *L. plantarum* CJLP 133 relative to the representative gut bacterium *Escherichia coli* when cultured in medium supplemented with SA, CNF, or SA/CNF composites (Figure 7A). The CNF samples achieved the highest PAS of 0.77 ± 0.07, followed by the 3% SA + 0.5% CNF composite (0.40 ± 0.12). In contrast, the pure SA samples exhibited the lowest score of 0.14 ± 0.03. Although the PAS of the SA/CNF composite was lower than that of the pure CNF, it represented an approximately 2.8-fold increase relative to the pure SA hydrogel. In particular, CNF not only enhanced the growth of probiotic *L. plantarum* CJLP 133 but also inhibited the growth of *E. coli*, indicating prebiotic effects.

Adhesion to gut epithelial cells (HT-29 cell lines) was also measured to determine whether SA/CNF hydrogel beads could facilitate the intestinal colonization of *L. plantarum* CJLP 133 (Figure 7B). The 3% SA + 0.5% CNF hydrogel exhibited an adhesion rate of 82.49% ± 1.27%, which was higher than the 74.10 ± 0.63% adhesion rate of the 3% SA hydrogel.

Nanocellulose has been widely studied as a novel prebiotic material because it is not degraded and absorbed in the human gut [72]. As only bacteria can use nanocellulose as a growth substrate, comparing the competitive inhibition of bacterial growth and intestinal adhesion is important for synbiotic applications [73]. The 3% SA + 0.5% CNF hydrogel promoted probiotic growth, inhibited pathogen growth, and enhanced the intestinal adhesion of probiotics, thereby confirming the synergistic effect of SA and CNF as prebiotic materials.

#### 2.4.2. Short-Chain Fatty Acid (SCFA) Production by *L. plantarum* CJLP 133 in SA/CNF Hydrogels

**Figure 8:**
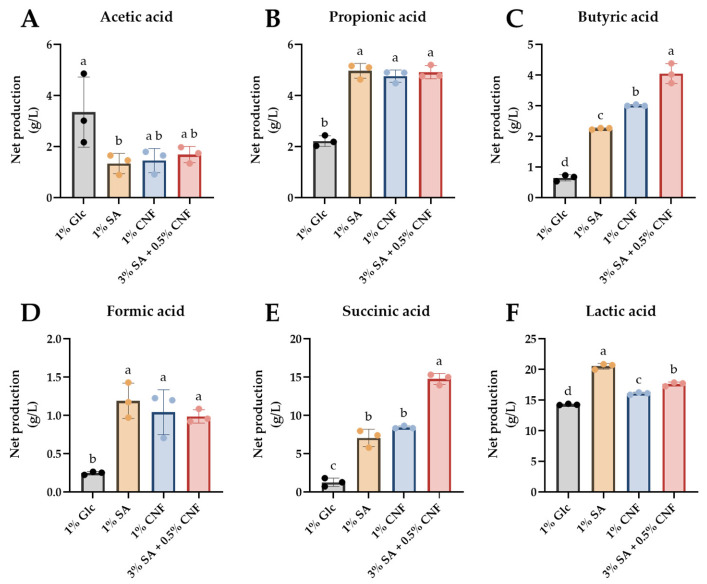
Net production of (**A**–**C**) short-chain fatty acids and (**D**–**F**) their precursor organic acids in *L. plantarum* CJLP 133 encapsulated with SA/CNF. Net production was calculated by subtracting the initial concentration (0 h) from the concentration measured after 24 h of fermentation. Data are presented as the mean ± SD (*n* = 3). Different lowercase letters indicate significant differences (*p* < 0.05).

A prebiotic matrix should serve as an effective carbon source that stimulates the production of SCFAs [74]. The production of representative SCFAs (e.g., acetic, propionic, and butyric acids) and their organic acid precursors (e.g., formic, succinic, and lactic acids) was examined in *L. plantarum* CJLP 133 cultured in each medium containing glucose, SA, CNF, or SA + CNF as the carbon source (Figure 8). Net production of SCFAs was calculated by subtracting the initial concentration at 0 h from the concentration after 24 h of cultivation. Although no significant correlation was noted between the precursors and SCFAs, a synergistic prebiotic effect of SA + CNF was observed in some SCFAs. Butyric acid production was most effectively increased using 3% SA + 0.5% CNF as the carbon source, reaching 4.05 ± 0.26 g/L. Acetic acid and propionic acid production showed no significant differences across the SA, CNF, and SA + CNF samples, ranging from 1.34 to 1.69 g/L and 4.76 to 4.97 g/L, respectively. In contrast, the levels of organic acid precursors varied distinctly depending on the carbon source. In particular, succinic acid, a precursor of propionic acid, reached the highest concentration (14.77 ± 0.58 g/L) in the SA + CNF composite. Although the production of formic acid (a precursor of acetic acid) and lactic acid (a precursor of propionic and butyric acids) was more effectively stimulated by SA or CNF alone than by the SA + CNF composite, the levels in the 3% SA + 0.5% CNF samples were 3.96- and 1.23-fold higher, respectively, than those in the 1% glucose control. These findings indicate that the SA/CNF composite produced SCFAs more efficiently than the standard 1% glucose control. Thus, *L. plantarum* CJLP 133 with the 3% SA + 0.5% CNF hydrogel exhibits potential as a novel synbiotic encapsulation product.

*L. plantarum* is known to ferment non-digestible polysaccharides through its endogenous cellulolytic enzymes, contributing to improved GI health [75,76]. The observed increase in both intestinal adhesion and SCFA production (e.g., butyrate and succinate) in the SA/CNF-hydrogel probably resulted from the enzymatic utilization of the matrix by *L. plantarum*. However, further research is required to fully elucidate the complex metabolism through which the SA/CNF composite is used by probiotics as a synergistic prebiotic source.

## 3. Conclusions

This study developed a novel synbiotic delivery system by incorporating CNF into an SA-based hydrogel matrix. The 3% SA + 0.5% CNF formulation demonstrated the greatest strategic advantage for probiotic encapsulation, thereby protecting viability under various processing conditions, such as heat shock, freeze-drying, low pH, and bile salts. This function is attributed to the intermolecular hydrogen bonding between SA and CNF, resulting in the formation of a denser network. Moreover, this hydrogel bead facilitated targeted and controlled release in the simulated GI system and provided prebiotic support to *L. plantarum* CJLP 133. These results suggest that the SA/CNF composite can be used as a promising platform for the stable and functional delivery of probiotics.

Although this study successfully confirms the in vitro efficacy of SA/CNF hydrogel beads, further mechanistic studies are required to understand their function within the complex human GI tract as a precision nutrition system. Also, the long-term storage stability of the encapsulated probiotics was not evaluated in this study. Further research focusing on the shelf-life and viability of SA/CNF hydrogel beads under various storage conditions is warranted to ensure their industrial feasibility.

## 4. Materials and Methods

### 4.1. Materials

CNFs (CNNT, Suwon, Republic of Korea), SA (Junsei, Tokyo, Japan), CaCl_2_ (Daejung, Siheung-si, Republic of Korea), and Lactobacilli De Man, Rogosa and Sharpe (MRS) broth (BD Difco, Franklin Lakes, NJ, USA) were used in this study. All media and reagents were sterilized at 121 °C for 15 min before use.

### 4.2. Preparation and Storage of Bacterial Culture

*L. plantarum* CJLP 133 was cultured in MRS broth at 37 °C for 24 h. The culture was centrifuged at 4 °C at 1971× *g* for 15 min, and the supernatant was then removed. The pellet was washed twice with 1× PBS (pH 7.4; Gibco, New York, NY, USA). For long-term storage, the bacterial suspension was mixed with 50% (*v*/*v*) glycerol at a 1:1 (*v*/*v*) ratio in sterile MRS broth and maintained as a stock at −80 °C.

### 4.3. Preparation of SA/CNF Hydrogel Beads

The encapsulation of *L. plantarum* CJLP 133 within the SA/CNF matrix was performed using an extrusion-based ionic gelation technique under sterile conditions. The encapsulation matrix was initially composed of various concentrations of SA stock solution (1%, 2%, and 3%, *w*/*v*) and CNF stock solution (0.5% and 1%, *w*/*v*). The bacterial pellet was thoroughly mixed with the SA/CNF solution at a 1:9 ratio (*v*/*v*). The resulting mixture was then extruded through a 21-gauge needle into 100 mL of sterile 0.1 M CaCl_2_ solution and allowed to harden for 30 min under continuous stirring [77]. To optimize the formulation, various SA:CNF mixing ratios (100:0, 80:20, 50:50, 40:60, 30:70, and 20:80) were evaluated. Based on the preliminary screening, the fixed volume ratio of 80:20 was selected for all subsequent experiments. The final concentrations of *L. plantarum* CJLP 133-loaded 3% SA + 0.5% CNF or 1% CNF (8:2) in the formulation were 2.16% and 0.18% or 0.09%, respectively. However, for convenience in sample labeling, the concentrations were expressed based on the stock solution concentrations (e.g., 3% SA, 3% SA + 0.5% CNF, and 3% SA + 1% CNF). The formed beads were collected, washed twice with sterile distilled water, and stored at 4 °C for further analysis. For each formulation condition, six independent *L. plantarum* CJLP 133-loaded beads were prepared, and the data were expressed as the mean ± standard deviation (*n* = 6).

### 4.4. Size Measurement and Texture Profile Analysis (TPA)

For size measurement, 10 capsules were randomly selected from each batch, and their diameters were measured using a digital caliper (Mitutoyo, Kawasaki, Japan). TPA was performed at room temperature (25 ± 2 °C) using a universal testing machine (ProLine Z010; Zwick Roell, Ulm, Germany), according to the protocol described in a previous research [78]. The beads were subjected to two compression cycles using a 35 mm diameter acrylic cylindrical probe with a 60 s relaxation time between compressions. Textural properties were determined using six independent replicates to ensure statistical reliability.

### 4.5. Morphological Characterization of SA/CNF Capsules

The surface and cross-sectional morphologies of the hydrogel beads were examined using field emission SEM (FE-SEM; SU8030, Hitachi, Tokyo, Japan). Before observation, the samples were sputter-coated with platinum (Pt) at 20 mA for 90 s to enhance electrical conductivity. The coated specimens were then analyzed using FE-SEM at a working distance of 11.8 mm and an accelerating voltage of 5 kV [79]. The pore sizes of SA/CNF hydrogel beads were quantitatively analyzed from SEM images using ImageJ 1.54g software (NIH, Bethesda, MD, USA). SEM images were converted to grayscale, and a threshold was set to distinguish pores from the hydrogel matrix. The pore diameters were measured using the particle analysis function, and more than 400 pores were analyzed for each sample after excluding the maximum and minimum values.

### 4.6. FT-IR Spectroscopic Analysis

The chemical structure and molecular interactions of the SA/CNF hydrogel beads were evaluated using FT-IR spectroscopy. Before analysis, the samples were freeze-dried for 48 h. Spectra were recorded using a Nicolet iS5 FT-IR spectrophotometer (Thermo Scientific, Waltham, MA, USA) equipped with an attenuated total reflectance (ATR) accessory (iD7; Thermo Scientific) featuring a zinc selenide (ZnSe) crystal. The spectra were acquired in ATR mode over a range of 550–4000 cm^−1^ at a resolution of 4 cm^−1^ with 16 scans per sample. Data processing and background correction were performed using OMNIC 9 software (Thermo Scientific) to eliminate atmospheric interference.

### 4.7. Encapsulation Efficiency of SA/CNF Capsules

To determine encapsulation efficiency, 1 g of the SA/CNF hydrogel bead sample was disintegrated in 9 mL of sterile 0.1 M sodium citrate buffer (Duksan, Ansan, Gyunggi-do, Republic of Korea) and vortexed for 10 min at room temperature to ensure the complete release of entrapped *L. plantarum* CJLP 133. The resulting suspension was serially diluted with sterile distilled water and plated on MRS agar. The plates were incubated at 37 °C for 48 h under anaerobic conditions. Encapsulation efficiency was calculated as follows, according to Equation (1):

Encapsulation Efficiency (%) = (log N/log N_0_) × 100 (1)

Here, N is the number of viable cells after encapsulation and N_0_ is the number before encapsulation.

### 4.8. Stability Assessment of SA/CNF Capsules

#### 4.8.1. Thermal Tolerance

To evaluate thermal stability, 1 g of each bead was placed in a test tube containing 10 mL of sterile distilled water. The tubes were exposed to various temperatures (50 °C, 70 °C, and 90 °C) for specific durations (15, 30, 0, 180, and 300 min) in a water bath. The temperature of the water bath was maintained at the setting temperature with a variance of ± 2 °C. Following heat treatment, the optical density (OD) was measured using UV–vis spectrophotometry (EPOCH; BioTek, Winooski, VT, USA). The survival rate (%) of both free and encapsulated probiotics was calculated using Equation (2), according to a previous study [70]:

Survival (%) = (OD_A_/OD_B_) × 100 (2)

Here, OD_A_ represents the value after heat treatment, while OD_B_ represents the value before heat treatment.

#### 4.8.2. Freeze-Drying Tolerance

Hydrogel beads were prefrozen at −50 °C for 24 h. Subsequently, the samples were lyophilized using a freeze-dryer (FreeZone 2.5 L Benchtop; Labconco, Kansas City, MO, USA) at −80 °C under a pressure of 0.007 mbar for 48 h. To assess viability, 1 g of the freeze-dried bead sample was disintegrated in 10 mL of sterile 0.1 M sodium citrate buffer by vortexing for 10 min [80]. The suspension was serially diluted in PBS, and 100 μL of each dilution was spread-plated onto MRS agar. The plates were incubated at 37 °C for 48 h, and the colony-forming units per gram (CFU/g) were then calculated using Equation (3):

Survival rate (%) = (log N/log N_0_) × 100 (3)

Here, N is the number of viable cells after freeze-drying (CFU/mL), while N_0_ is the number of initial viable cells (CFU/mL).

### 4.9. Acid and Bile Tolerance

The acid and bile tolerance capacities of encapsulated *L. plantarum* CJLP 133 were evaluated using a previously reported method [70], with slight modifications. In brief, 1 g of the SA/CNF hydrogel bead sample was suspended in 10 mL of PBS solution. To determine acid tolerance, the bacterial suspensions were inoculated into 10 mL of MRS broth, which had been adjusted to pH 2.5 using 6 N hydrochloric acid (HCl), and incubated at 37 °C for 2 h. To determine bile tolerance, the samples were inoculated into MRS broth containing 0.3% (*w*/*v*) of bile salt (ox gall/ox bile) and incubated at 37 °C for 3 h. After each incubation period, the beads were disintegrated in 0.1 M sodium citrate buffer to release the entrapped cells. The survival rate (%) against acid and bile salts was calculated using Equation (4):

Survival rate (%) = (log N/log N_0_) × 100 (4)

Here, N is the number of viable cells against acid or bile salts (CFU/mL), while N_0_ is the number of initial viable cells (CFU/mL).

### 4.10. In Vitro Release Profiles

#### 4.10.1. Preparation of SGF and SIF

Simulated gastric fluid (SGF) was prepared by dissolving 9 g of sodium chloride (NaCl; Duksan) in 1000 mL of sterile water, adjusting the pH to 2.0 with 1 M HCl solution (Duksan), and then dissolving 0.3% pepsin (Pepsin 1:10,000; Wako, Osaka, Japan) in it. Simulated intestinal fluid (SIF) was prepared using KH_2_PO_4_ (Duksan) containing 1% bile salt (ox gall/ox bile) and 1% pancreatin (Kanto, Tokyo, Japan), with the pH adjusted to 7.4. The compositions of SGC and SIF were prepared based on previously reported simulated GI conditions [81,82].

#### 4.10.2. Release Behavior

SGF and SIF were used to assess the release behavior of *L. plantarum* CJLP 133 from SA/CNF hydrogel beads. The release experiment was conducted with reference to the reported method [83]. In brief, 1 g of the wet bead sample was immersed in 9 mL of SGF and incubated at 37 °C for 2 h. After the gastric phase, the beads were recovered via filtration, washed twice with sterile distilled water, and then transferred into 9 mL of SIF for further incubation at 37 °C. Samples were collected at 0, 60, and 120 min during the SGF phase and at 0, 30, 60, 90, 120, and 180 min during the SIF phase. At each sampling interval, the viable cell counts were determined as described in Section 4.7. For viable cell count, the hydrogel beads were dissolved using a sodium citrate buffer to release encapsulated cells prior to plating [80]. To maintain a constant volume throughout the experiment, 1 mL of the supernatant was withdrawn for analysis and immediately replaced with 1 mL of fresh, sterile SIF.

### 4.11. Prebiotic Activity Assay

To determine the ability of the SA/CNF matrix to selectively stimulate the growth of probiotics in comparison with enteric bacteria, the PAS was measured according to a previously described method [84]. *L. plantarum* CJLP 133 was used as the probiotic strain, and *E. coli* KCTC 2571 was used as the enteric strain. The basal medium used was MRS broth without glucose (MB Cell, Seoul, Republic of Korea), which was supplemented with a prebiotic candidate, such as SA, CNF, or SA + CNF. Each strain was inoculated into the prepared media and incubated at 37 °C for 24 h. The viable cell counts of the probiotic strain were determined by plating on MRS agar, while those of the enteric strain were determined by plating on LB agar (BD Difco). The prebiotic score (P.S.) was calculated using Equation (5):

P.S. = (A − B)/(C − D)(5)

Here, A represents the bacterial population (log CFU/mL) grown on the prebiotic at 24 h, B represents the bacterial population (log CFU/mL) grown on the prebiotic at 0 h, C represents the bacterial population (log CFU/mL) grown on glucose at 24 h, and D represents the bacterial population (log CFU/mL) grown on glucose at 0 h.

### 4.12. Assessment of Adhesion to Gut Epithelial Cells

The gut adhesion ability of *L. plantarum* CJLP 133 was evaluated using the HT-29 human colorectal adenocarcinoma cell line, as described previously [70]. HT-29 cells were purchased from the Korean Cell Line Bank (KCTC; Seoul, Republic of Korea) and cultured in DMEM supplemented with 10% FBS and 1% penicillin/streptomycin at 37 °C in a 5% CO_2_ incubator and seeded in six-well plates at a density of 1 × 10^6^ cells/mL for 24 h. Before the assay, the SA/CNF hydrogel beads were sequentially exposed to SGF for 2 h and SIF for 3 h. The released bacteria were then harvested and resuspended in antibiotic-free DMEM at a concentration of 1 × 10^5^ CFU/mL. Subsequently, the bacterial suspension was added to the HT-29 cells and incubated for 45 min at 37 °C. After incubation, nonadherent bacteria were removed by washing with sterile PBS, and the cells with adherent bacteria were detached using a trypsin–EDTA solution. The concentration of attached bacteria was quantified by serial dilution and plating on MRS agar, and the adhesion rate (%) was calculated as the ratio of attached bacteria to the initial bacterial population based on three independent replicates.

### 4.13. SCFA Analysis

To evaluate the production of SCFAs, *L. plantarum* CJLP 133 was cultured in glucose-free MRS broth (MB Cell) supplemented with either 1% (*w*/*v*) glucose (as a control) or 1% (*w*/*v*) of various prebiotic candidates (SA and CNF). This approach, involving the substitution of glucose with alternative carbon sources to assess microbial metabolism, was adopted based on previously reported methods [85]. The strain was inoculated at a 1% (*v*/*v*) ratio and incubated at 37 °C for 24 h. Subsequently, the culture broth was centrifuged at 1971× *g* for 15 min at 4 °C to obtain the supernatant. The supernatant was filtered through a 0.22-μm hydrophobic PTFE membrane filter (Futecs, Daejeon, Republic of Korea). SCFA analysis was performed using a high-performance liquid chromatography (HPLC) system (LC-6000; Futecs) equipped with an Aminex HPX-87H organic column (Bio-Rad, Hercules, CA, USA). The mobile phase consisted of 0.005 M H_2_SO_4_ at a flow rate of 0.5 mL/min. The concentration of individual SCFAs was quantified based on standard curves. The HPLC-based analysis of SCFAs was conducted following the previous methods [86].

### 4.14. Data Analysis

All experiments were conducted at least in triplicate. The results are expressed as the mean ± standard deviation (SD). Statistical analysis was performed using SPSS software (version 21.0; IBM Corp., Armonk, NY, USA) and GraphPad Prism (version 11.0; GraphPad Software Inc., San Diego, CA, USA). Statistical significance of the differences between groups was determined using one-way analysis of variance (ANOVA), followed by Tukey’s multiple range tests. A *p*-value of <0.05 was considered to indicate a statistically significant difference.

## Figures and Tables

**Table 1:** Diameter (mm) of SA/CNF hydrogel beads prepared with different concentrations and ratios of SA and CNF. Data are presented as the mean ± SD (*n* = 6). Different uppercase letters indicates statistically significant differences among all columns, and different lowercase letters indicates statistically significant differences among all rows (*p* < 0.05).

	Size (mm)							
Ratio SA:CNF	CaCl_2_		0.1 M	0.5 M	0.1 M	0.5 M	0.1 M	0.5 M
		* SA	1%		2%		3%	
	** CNF							
100:0	1%		1.80 ± 0.05 ^aC^	1.76 ± 0.05 ^aD^	2.26 ± 0.04 ^bF^	2.25 ± 0.02 ^bF^	2.55 ± 0.02 ^dG^	2.47 ± 0.02 ^cF^
80:20			1.78 ± 0.06 ^bC^	1.57 ± 0.05 ^aC^	2.25 ± 0.02 ^cEF^	2.23 ± 0.06 ^cEF^	2.45 ± 0.04 ^eF^	2.36 ± 0.08 ^dE^
50:50			1.62 ± 0.03 ^bB^	1.24 ± 0.08 ^aB^	2.23 ± 0.03 ^cE^	2.19 ± 0.07 ^cDE^	2.33 ± 0.03 ^dE^	2.24 ± 0.03 ^cD^
40:60			*** -	-	2.17 ± 0.02 ^bcD^	2.15 ± 0.04 ^bD^	2.30 ± 0.04 ^dDE^	2.20 ± 0.04 ^cD^
30:70			-	-	-	-	2.21 ± 0.05 ^cC^	2.15 ± 0.03 ^bC^
20:80			-	-	-	-	2.13 ± 0.06 ^bB^	2.10 ± 0.07 ^bC^
80:20	0.5%		1.85 ± 0.02 ^bD^	1.79 ± 0.07 ^aD^	2.25 ± 0.03 ^Def^	2.19 ± 0.04 ^cDE^	2.44 ± 0.02 ^eF^	2.45 ± 0.03 ^eF^
50:50			-	-	2.10 ± 0.05 ^cC^	1.94 ± 0.05 ^bC^	2.33 ± 0.10 ^eE^	2.21 ± 0.05 ^dD^
40:60			-	-	1.96 ± 0.02 ^cB^	1.89 ± 0.03 ^bB^	2.27 ± 0.02 ^eCD^	2.12 ± 0.05 ^dC^
30:70			-	-	-	-	2.06 ± 0.02 ^cA^	1.87 ± 0.04 ^bB^
20:80			-	-	-	-	2.01 ± 0.05 ^bA^	1.60 ± 0.02 ^bA^

* SA: Sodium alginate; ** CNF: Cellulose nanofibers; *** -: No discrete beads were formed; the formation resulted in a spread-out mass.

**Table 2:** Texture profile analysis (TPA) of SA/CNF hydrogel beads. Data are presented as the mean ± SD (*n* = 6). Different lowercase letters indicate significant differences (*p* < 0.05).

Sample	Parameter					
	Hardness (N)	Adhesiveness (mJ)	Springiness	Gumminess (N)	Chewiness (N)	Cohesiveness
3% SA	8.80 ± 0.29 ^a^	0.01 ± 0.01 ^a^	0.79 ± 0.02 ^a^	4.70 ± 0.29 ^a^	3.73 ± 0.31 ^a^	0.53 ± 0.02 ^a^
3% SA + 0.5% CNF	7.15 ± 0.23 ^b^	0.01 ± 0.00 ^a^	0.77 ± 0.02 ^ab^	3.69 ± 0.16 ^b^	2.85 ± 0.16 ^b^	0.52 ± 0.01 ^ab^
3% SA + 1% CNF	7.18 ± 0.15 ^b^	0.01 ± 0.00 ^a^	0.79 ± 0.00 ^a^	3.80 ± 0.07 ^b^	3.00 ± 0.06 ^b^	0.53 ± 0.01 ^a^

**Table 3:** Encapsulation efficiency (%) of *L. plantarum* CJLP 133 with SA/CNF hydrogel beads. Data are presented as the mean ± SD (*n* = 3). Different lowercase letters indicate significant differences (*p* < 0.05).

*L. plantarum* CJLP 133	Log (CFU/mL)	Encapsulation Efficiency (%)
Non coated	8.72 ± 0.17 ^a^	-
3% SA	8.38 ± 0.05 ^b^	96.12 ± 0.57 ^b^
3% SA + 0.5% CNF	8.46 ± 0.04 ^b^	97.01 ± 0.45 ^b^
3% SA + 1% CNF	8.35 ± 0.01 ^b^	95.73 ± 0.17 ^b^

## Data Availability

Data will be made available on request.

## Abbreviations

The following abbreviations are used in this manuscript:

SA	Sodium alginate
CNF	Cellulose nanofiber
SD	Standard deviation
SEM	Scanning electron microscopy
TPA	Texture profile analysis
FT-IR	Fourier transform infrared spectroscopy
GI	Gastrointestinal
SGF	Simulated gastric fluid
SIF	Simulated intestinal fluid
3D	Three-dimensional
Ca^2+^	Calcium ion
CaCl_2_	Calcium chloride anhydride
COO	Carboxyl group
EE	Encapsulation efficiency
CFU	Colony-forming units
PAS	Prebiotic activity score
SCFAs	Short-chain fatty acids
e.g.,	Exempli gratia
MRS	Lactobacilli De Man, Rogosa and Sharpe
PBS	Phosphate-buffered saline
FE-SEM	Field emission-scanning electron microscopy
ATR	Attenuated total reflectance
ZnSe	Zinc selenide
OD	Optical density
UV	Ultraviolet
HPLC	High-performance liquid chromatography
PTFE	Polytetrafluoroethylene
ANOVA	Analysis of variance
